# Supplementary material for: Discovery and application of insertion-deletion (INDEL) polymorphisms for QTL mapping of early life-history traits in Atlantic salmon
Source: BMC Genomics. 2010 Mar 8;11:156. doi: 10.1186/1471-2164-11-156 (PMC2838853; doi:10.1186/1471-2164-11-156)
Supplement: Additional file 2 — Information on developed 76 locus single-run INDEL panel in Atlantic salmon. Information on fluorescence labeling, primer concentrations, PCR pooling and links to alignments, INDEL motifs and GENESCAN (Burge and Karlin 1997) predictions of genes/exons are available in html format. [file 1471-2164-11-156-S2.ZIP › Additionalfile2/snpsummary4883.html]

```
Cluster 1187 Contig 1

prev  Summary    Contig List  next
```

Size of Consensus sequence = 2203

Number of sequences = 29

Minimum redundancy = 6

Key

A gi|118419820|gb|AM412202.1|AM412202 AM412202 Salmo salar after live bacterial vaccine Salmo salar cDNA clone kid\_aki\_03D05, mRNA sequence  
B gi|117844986|gb|EG917682.1|EG917682 EST\_ssal\_evf\_56811 ssalevf mixed\_tissue Salmo salar cDNA Salmo salar cDNA clone ssal\_evf\_576\_073\_fwd 3', mRNA sequence  
C gi|117514296|gb|EG846055.1|EG846055 EST\_ssal\_eve\_41 ssaleve thyroid Salmo salar cDNA Salmo salar cDNA clone ssal\_eve\_001\_024\_fwd 3', mRNA sequence  
D gi|57125252|gb|CX356693.1|CX356693 ssalrgb522238\_rev\_0 mixed\_tissue Salmo salar cDNA, mRNA sequence  
E gi|117504644|gb|EG836403.1|EG836403 EST\_ssal\_eve\_11053 ssaleve thyroid Salmo salar cDNA Salmo salar cDNA clone ssal\_eve\_515\_005\_fwd 3', mRNA sequence  
F gi|89862769|gb|DY718892.1|DY718892 EST\_ssal\_rgb2\_74631 ssalrgb2 mixed\_tissue Salmo salar cDNA Salmo salar cDNA clone ssal\_rgb2\_622\_291\_rev 5', mRNA sequence  
G gi|85027333|gb|DW555989.1|DW555989 EST\_ssal\_rgb2\_20408 rgb2 Salmo salar cDNA clone ssal\_rgb2\_533\_115\_rev 5', mRNA sequence  
H gi|45304001|gb|CK874370.1|CK874370 SGP135576 Atlantic salmon Brain cDNA library Salmo salar cDNA clone HJ4-0375 5', mRNA sequence  
I gi|85034909|gb|DW563565.1|DW563565 EST\_ssal\_rgb2\_27984 rgb2 Salmo salar cDNA clone ssal\_rgb2\_545\_175\_rev 5', mRNA sequence  
J gi|45304079|gb|CK874448.1|CK874448 SGP135657 Atlantic salmon Brain cDNA library Salmo salar cDNA clone HJ4-0475 5', mRNA sequence  
K gi|50134842|gb|CO469826.1|CO469826 SGP262354 Atlantic salmon Eye cDNA library Salmo salar cDNA clone OY4-1186 5', mRNA sequence  
L gi|85026801|gb|DW555457.1|DW555457 EST\_ssal\_rgb2\_19876 rgb2 Salmo salar cDNA clone ssal\_rgb2\_532\_186\_rev 5', mRNA sequence  
M gi|85049779|gb|DW577957.1|DW577957 EST\_ssal\_rgb2\_42376 rgb2 Salmo salar cDNA clone ssal\_rgb2\_568\_255\_fwd 3', mRNA sequence  
N gi|24390382|gb|CA060139.1|CA060139 ssalrgb513069 mixed\_tissue Salmo salar cDNA, mRNA sequence  
O gi|24389892|gb|CA059649.1|CA059649 ssalrgb522238 mixed\_tissue Salmo salar cDNA, mRNA sequence  
P gi|29325548|gb|CB514322.1|CB514322 ssalrgb552020 mixed\_tissue Salmo salar cDNA, mRNA sequence  
Q gi|117504645|gb|EG836404.1|EG836404 EST\_ssal\_eve\_11054 ssaleve thyroid Salmo salar cDNA Salmo salar cDNA clone ssal\_eve\_515\_005\_rev 5', mRNA sequence  
R gi|24344963|gb|CA043992.1|CA043992 ssalpha501175 gut Salmo salar cDNA, mRNA sequence  
S gi|24355707|gb|CA049537.1|CA049537 ssalkhc003074 kidney Salmo salar cDNA, mRNA sequence  
T gi|85027334|gb|DW555990.1|DW555990 EST\_ssal\_rgb2\_20409 rgb2 Salmo salar cDNA clone ssal\_rgb2\_533\_115\_fwd 3', mRNA sequence  
U gi|29310710|gb|CB499484.1|CB499484 ssalkhc013035 kidney Salmo salar cDNA, mRNA sequence  
V gi|24392588|gb|CA062345.1|CA062345 ssalrga509366 mixed\_tissue Salmo salar cDNA, mRNA sequence  
W gi|85034910|gb|DW563566.1|DW563566 EST\_ssal\_rgb2\_27985 rgb2 Salmo salar cDNA clone ssal\_rgb2\_545\_175\_fwd 3', mRNA sequence  
X gi|117844987|gb|EG917683.1|EG917683 EST\_ssal\_evf\_56812 ssalevf mixed\_tissue Salmo salar cDNA Salmo salar cDNA clone ssal\_evf\_576\_073\_rev 5', mRNA sequence  
Y gi|117842494|gb|EG915190.1|EG915190 EST\_ssal\_evf\_54566 ssalevf mixed\_tissue Salmo salar cDNA Salmo salar cDNA clone ssal\_evf\_573\_050\_rev 5', mRNA sequence  
Z gi|24343314|gb|CA042409.1|CA042409 ssalplnb501108 gut Salmo salar cDNA, mRNA sequence  
a gi|85026800|gb|DW555456.1|DW555456 EST\_ssal\_rgb2\_19875 rgb2 Salmo salar cDNA clone ssal\_rgb2\_532\_186\_fwd 3', mRNA sequence  
b gi|89862768|gb|DY718891.1|DY718891 EST\_ssal\_rgb2\_74630 ssalrgb2 mixed\_tissue Salmo salar cDNA Salmo salar cDNA clone ssal\_rgb2\_622\_291\_fwd 3', mRNA sequence  
c gi|117514295|gb|EG846054.1|EG846054 EST\_ssal\_eve\_40 ssaleve thyroid Salmo salar cDNA Salmo salar cDNA clone ssal\_eve\_001\_024\_rev 5', mRNA sequence

4 SNPs detected

A B C D E F G H I J K L M N O P Q R S T U V W X Y Z a b c  cosegregation weighted

2084 . . . . . . . . . . . . . - - - - - - - A A - - - A A A A   4/4 55.17
2085 . . . . . . . . . . . . . - - - - - - - G G - - - G G G G   4/4 55.17
2086 . . . . . . . . . . . . . - - - - - - - T T - - - T T T T   4/4 55.17
2087 . . . . . . . . . . . . . - - - - - - - A A - - - A A A A   4/4 55.17
